# Incidence of metallo-beta-lactamase producing clinical isolates of *Escherichia coli* and *Klebsiella pneumoniae* in central Nepal

**DOI:** 10.1186/1756-0500-7-557

**Published:** 2014-08-21

**Authors:** Arijit Bora, Rajkumari Sanjana, Brajesh Kumar Jha, Surya Narayan Mahaseth, Khilasa Pokharel

**Affiliations:** Department of Microbiology, College of Medical Sciences, P.O. Box 23, Bharatpur, Nepal

**Keywords:** *Escherichia coli*, *Klebsiella pneumoniae*, Metallo-beta-lactamase, Carbapenemase, Multidrug-resistant, Pandrug-resistant

## Abstract

**Background:**

The increasing and rapid spread of metallo-beta-lactamase (MBL) producing *Enterobacteriaceae*, particularly *Escherichia coli* and *Klebsiella pneumoniae* represents an emerging public health threat. However, limited data is available on MBL production in clinical isolates of *E. coli* and *K. pneumoniae* from Nepal. We have therefore undertaken this study to ascertain the incidence of MBL production in clinical isolates of *E. coli* and *K. pneumoniae* at a tertiary care teaching hospital in central Nepal.

**Methods:**

A total of 401 consecutive, non-duplicate isolates of *E. coli* (n = 216) and *K. pneumoniae* (n = 185) were recovered from various clinical samples between July and December, 2012. These isolates were screened for the detection of carbapenemase production on the basis of their reduced susceptibility to meropenem or ertapenem by the disc diffusion method. The screened isolates were further phenotypically studied for carbapenemase production by modified Hodge test (MHT). MBL production was detected by performing combined disc test by using imipenem discs with and without ethylenediaminetetraacetic acid (EDTA), which chelates zinc required for MBL activity.

**Results:**

Out of 216 *E. coli* isolates, a total of 41 isolates (18.98%) and out of 185 *K. pneumoniae* isolates, a total of 39 isolates (21.08%) were suspected to be carbapenemase- producers on the basis of their reduced susceptibility to meropenem or ertapenem. Interestingly, all the initially suspected isolates of *E. coli* and *K. pneumoniae* for carbapenemase production were found to be positive in both MHT and combined disc test. However, few weakly positive reactions were observed in MHT. All the MBL producing isolates were multidrug-resistant (MDR). In addition, 75.60% *E. coli* and 71.79% of *K. pneumoniae* isolates producing MBL were found to be “pandrug- resistant”.

**Conclusions:**

Our findings showed MBL production in a considerable number of *E. coli* and *K. pneumoniae* isolates with MDR and pandrug-resistant phenotypes. Combined disc method can provide a sensible choice for phenotypic detection of MBL production in clinical microbiology laboratories as detection of MBL in bacterial isolates is indispensable for establishing the effective antibiotic policies and infection control strategies in the hospital setting.

## Background

Over the past few years, metallo-beta-lactamase (MBL) producing gram negative bacteria are being reported with increasing frequency from several parts of the world and have emerged as a most widespread and clinically significant carbapenem resistance mechanisms [1]. MBL producing bacteria can hydrolyze a wide range of beta-lactam antibiotics including penicillins, cephalosporins, carbapenems, cephamycins, but lack the ability to hydrolyze aztreonam. Moreover, their catalytic activities are generally not neutralized by commercially available β-lactamase inhibitors such as clavulanate, tazobactam, and sulbactam [2]. These enzymes belong to Ambler class B beta-lactamases based on their amino acid sequence homology and to group 3 according to the Bush classification based on their substrate and inhibitor profiles [3, 4]. MBLs require zinc-ions to catalyze the hydrolysis of beta-lactam antibiotics and due to the dependence on zinc-ions, MBL catalysis is inhibited in presence of metal-chelating agents like ethylenediaminetetraacetic acid (EDTA) [5].

MBLs are encoded either by genes that are part of the chromosome in some bacterial species (resident MBLs), or by heterologous genes acquired by horizontal gene transfer (acquired MBLs) [6]. The more geographically widespread MBLs include imipenemase (IMP), Verona integron-encoded metallo-beta-lactamase (VIM), and New Delhi metallo-beta-lactamase (NDM) [5]. MBLs were common in *Pseudomonas aeruginosa* and *Acinetobacter* spp., but more recently have emerged at an increasing rate among the members of *Enterobacteriaceae* [7].

Although, production of MBLs in clinical isolates represent a serious therapeutic challenge, till date clinical data are surprisingly inadequate with regard to their incidence in the members of *Enterobacteriaceae*, particularly from Nepal. This prompted us to conduct the present study to determine the incidence of MBL production phenotypically among the isolates of two important members *of Enterobacteriaceae*, *Escherichia coli* and *Klebsiella pneumoniae* as well as their antibiotic susceptibility pattern to formulate an antimicrobial policy on the basis of the local epidemiological data.

## Methods

### Bacterial isolates

A total of 401 consecutive, non-duplicate isolates of *E. coli* (n = 216) *and K. pneumoniae* (n = 185) were recovered from various clinical samples in clinical microbiology laboratory of College of Medical Sciences, a tertiary care 1050-bed teaching hospital in central Nepal. Samples were obtained from the hospitalized patients of different hospital units between July and December, 2012. Distribution of the sources of these isolates was: urine (n = 275), sputum (n = 62), pus (n = 39) and blood (n = 25). The samples were processed for isolation and identification based on standard laboratory techniques [8]. This study was carried out with consent from the Institutional Review Committee (IRC) of College of Medical Sciences, Bharatpur, Nepal.

### Antimicrobial susceptibility testing

Antimicrobial sensitivity testing was performed on Mueller-Hinton agar (MHA) plates by Kirby-Bauer disc diffusion method, according to Clinical Laboratory Standards Institute (CLSI) guidelines [9]. The antibiotics used were: ampicillin (10 μg), cephalexin (30 μg), cefotaxime (30 μg), ceftazidime (30 μg), cefpodoxime (10 μg), ceftriaxone (30 μg), cefepime (30 μg), aztreonam (30 μg), cefoxitin (30 μg), piperacillin/tazobactam (100/10 μg), imipenem (10 μg), meropenem (10 μg), ertapenem (10 μg), co-trimoxazole (25 μg), ciprofloxacin (5 μg), levofloxacin (5 μg), gentamicin (10 μg), amikacin (30 μg), tigecycline (15 μg) and colistin (10 μg). All the antibiotic discs and the media were procured from Hi-media, Mumbai, India. *E. coli* ATCC 25922 was used as quality controls in antibiotic susceptibility testing. The results were interpreted as per CLSI guidelines [9] except, tigecycline and colistin. The results for colistin were interpreted by following the criteria proposed by Galani et al. [10] and for tigecycline by following the breakpoints for *Enterobacteriaceae* as suggested by Food and Drug Administration (FDA). The minimum inhibitory concentration (MIC) values for imipenem, meropenem, ertapenem, tigecycline and colistin were determined by using Etest strips (bioMerieux, France) as per the manufacturer’s protocol.

### Screening for carbapenemase production

By disc diffusion, each of the isolate with a reduced susceptibility to meropenem or ertapenem (inhibition zone diameter of ≤21 mm) was screened for the production of carbapenemase according to the CLSI guidelines [9].

### Detection of carbapenemase production

**Figure 1:**
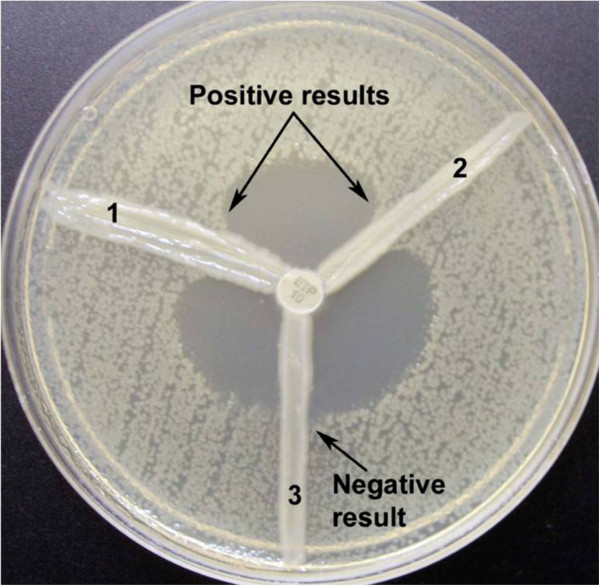
**Modified Hodge test using a 10 μg ertapenem disc.** Isolates 1 and 2 produce carbapenemase and are positive by this test. Isolate 2 does not produce carbapenemase and is negative by this test.

The phenotypic detection of the carbapenemase production was performed by the modified Hodge test (MHT) as described by CLSI [9]. Briefly, a 0.5 McFarland standard suspension of *E. coli* ATCC 25922 was prepared in 5 ml peptone water and diluted 1:10 by adding 0.5 ml of the 0.5 McFarland to 4.5 ml of peptone water. A lawn of the 1:10 dilution of *E. coli* ATCC 25922 was prepared on a MHA plate as for the routine disc diffusion procedure. The plate was allowed to dry 3 to 10 minutes. A 10 μg ertapenem disc was placed in the centre of the test plate and the test organism was streaked in a straight line from the edge of the disc to the edge of the plate. Three organisms were tested on the same plate with one drug. The plate was incubated at 37°C in ambient air for 16–24 hours. After incubation, a positive MHT test was indicated by a clover leaf-like indentation of the *E. coli* ATCC 25922 growing along the test organism growth streak within the disc diffusion zone and a negative MHT test was indicated by no growth of the *E. coli* ATCC 25922 along the test organism growth streak within the disc diffusion zone (Figure 1).

### Detection of metallo-beta-lactamase production

**Figure 2:**
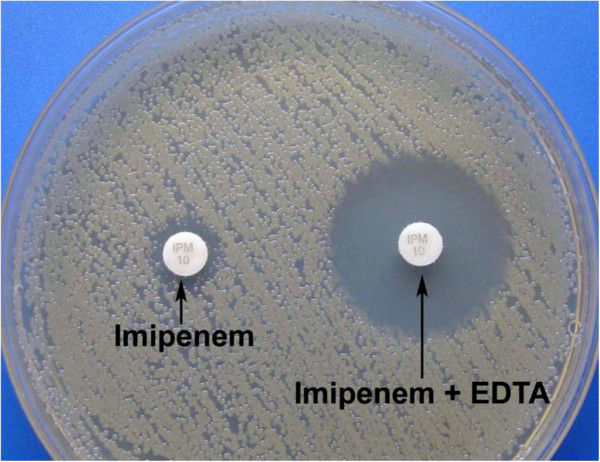
**Positive combined disc test for detection of MBL producer by use of EDTA.** The test isolate shows a zone diameter of >4 mm around the imipenem-EDTA disc compared to that of the imipenem disc alone.

MBL production was detected by performing combined disc test described by Franklin et al. [11] in all carbapenemase screening positive isolates. In this test, two imipenem discs (10 μg), one containing 10 μl of 0.1 M (292 μg) anhydrous EDTA (Sigma Chemicals, St. Louis, MO) were used. They were placed on a MHA plate inoculated with 0.5 McFarland suspension of the test isolate. Plates were incubated for 16–18 hours at 35°C. After incubation, the diameter of inhibition zones was measured. An increase in zone diameter of >4 mm around the imipenem-EDTA disc compared to that of the imipenem disc alone was considered positive for MBL production (Figure 2).

*E. coli* strain NDM-1 EC27 (positive for NDM-1; kindly provided by Prof. Giasuddin Ahmed) was used as positive control in MHT and combined disc test. *E. coli* ATCC 25922 was used as negative control for both the tests.

## Results

Out of 216 *E. coli* isolates, a total of 41 isolates (18.98%) and out of 185 *K. pneumoniae* isolates, a total of 39 isolates (21.08%) were initially screened for carbapenemase production on the basis of their reduced susceptibility to meropenem or ertapenem by disc diffusion test. All the screening positive isolates of *E. coli* and *K. pneumoniae* were found to be positive for carbapenemase production by MHT. However, 3 (7.31%) isolates of *E. coli* and 4(10.26%) isolates of *K. pneumoniae* gave weakly positive reactions in MHT. Interestingly, all the screening positive isolates of *E. coli* and *K. pneumoniae* showed fairly positive results in combined disc test for MBL production. The age of the patients with MBL positive isolates ranged from 10 days to 72 years and the male to female ratio was 1.15:1.

**Figure 3:**
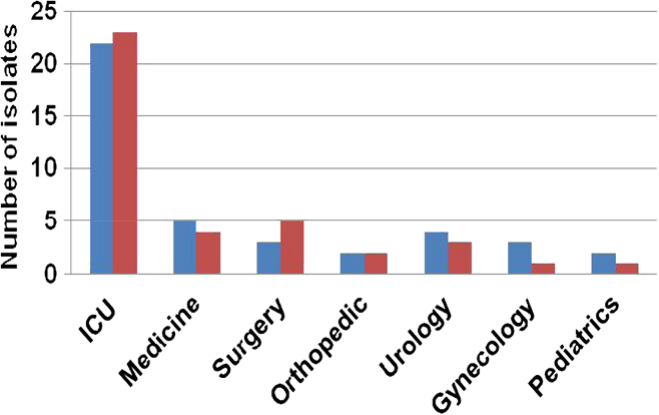
**Distribution of MBL producing isolates of** ***E. coli*** **and** ***K. pneumoniae*** **in different hospital units.**

**Figure 4:**
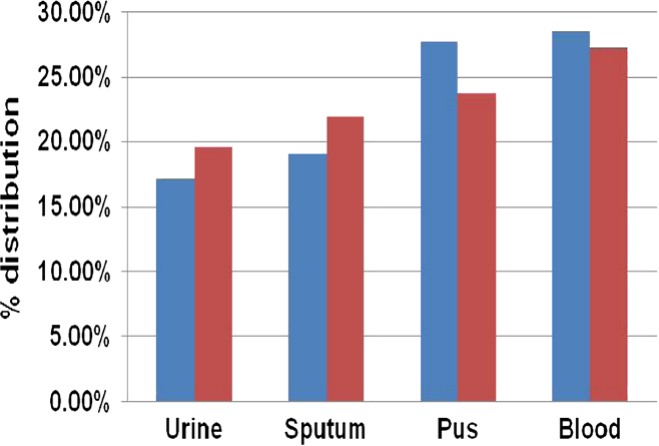
**Sample-wise distribution of MBL producing isolates of** ***E. coli*** **and** ***K. pneumoniae*** **.**

Among the MBL positive isolates of *E. coli*, 53.56% (22/41) isolates were recovered from the patients admitted to intensive care units (ICU) and 46.34% (19/41) isolates were recovered from the patients admitted to different hospital wards (Figure 3). Likewise, among the MBL positive isolates of *K. pneumoniae*, 58.97% (23/39) isolates were recovered from the patients admitted to ICU and 46.15% (18/39) isolates were recovered from the patients admitted to different hospital wards (Figure 3). Sample-wise distribution of MBL producing isolates of *E. coli* and *K. pneumoniae* are shown in Figure 4.

By disc diffusion susceptibility testing, each of the MBL producing isolate of *E. coli* and *K. pneumoniae* was found to be resistant to all beta-lactam antibiotics including imipenem, meropenem, ertapenem, aztreonam as well as beta-lactam/beta-lactamase combination used in this study. Susceptibility pattern of these isolates differed with different classes of non- beta-lactam antibiotics (Table 1). All the MBL producing isolates are found to be multidrug-resistant (MDR) i.e., resistant to three or more classes of antimicrobials. In addition, 75.60% of MBL producing *E. coli* isolates and 71.79% of *K. pneumoniae* MBL producing *E. coli* isolates were “pandrug- resistant” i.e., resistant to 7 antimicrobial agents (cefepime, ceftazidime, imipenem, meropenem, piperacillin/tazobactam, ciprofloxacin, and levofloxacin). However, each of the MBL producing isolate of *E. coli* and *K. pneumoniae* were found to be sensitive to tigecycline and colistin by disc diffusion test. The MIC values of MBL producing *E. coli* and *K. pneumoniae* isolates were found to vary widely for the different carbapenems used in the study (Table 2). All MBL producing isolates showed MIC values in effective range for tigecycline and colistin.

**Table 1:** **In vitro susceptibility pattern of MBL producing and non-MBL-producing isolates of** ***E. coli*** **and** ***K. pneumoniae*** **by disc diffusion test**

Antibiotics tested	% sensitive			
	***E. coli***(n = 216)		***K. pneumoniae***(n = 185)	
	Non-MBL producer (n = 175)	MBL producer (n = 41)	Non-MBL producer (n = 146)	MBL producers (n = 39)
Ampicillin (10 μg)	2.85	0.00	0.00	0.00
Cephalothin (30 μg)	16.57	0.00	13.69	0.00
Cefpodoxime (10 μg)	35.42	0.00	33.56	0.00
Ceftazidime (30 μg)	40.57	0.00	37.67	0.00
Cefotaxime (30 μg)	38.85	0.00	39.72	0.00
Ceftriaxone (30 μg)	43.42	0.00	42.46	0.00
Cefepime (30 μg)	46.85	0.00	44.52	0.00
Aztreonam (30 μg)	40.0	0.00	41.09	0.00
Cefoxitin (30 μg)	64.57	0.00	69.18	0.00
Piperacillin/tazobactam (100/10 μg)	82.85	0.00	77.39	0.00
Imipenem (10 μg)	100.00	0.00	100.00	0.00
Meropenem (10 μg)	100.00	0.00	100.00	0.00
Ertapenem (10 μg)	100.00	0.00	100.00	0.00
Ciprofloxacin (5 μg)	64.00	7.31	67.12	10.25
Levofloxacin (5 μg)	85.14	24.39	87.67	28.20
Co-Trimoxazole	61.14	0.00	65.75	7.69
Gentamicin (10 μg)	72.57	17.07	70.54	15.38
Amikacin (30 μg)	80.57	21.95	78.08	20.51
Tigecycline (15 μg)	100.00	100.00	100.00	100.00
Colistin (10 μg)	100.00	100.00	100.00	100.00

**Table 2:** **MIC ranges found for MBL producing** ***E. coli*** **and** ***K. pneumoniae*** **isolates by using Etest strips**

MBL producing isolates	MIC range (μg/mL)				
	Imipenem	Meropenem	Ertapenem	Tigecycline	Colistin
*E. coli*	2.0 - 8.0	3.0 - 16	8.0- > 32	0.125 - 0.75	0.125 - 0.5
*K. pneumoniae*	2.0- >32	2.0- >32	6.0- >32	0.38 - 2.0	0.125 - 0.5

## Discussion

The increasing and rapid spread of MBL producing *Enterobacteriaceae*, particularly *E. coli* and *K. pneumoniae* constitutes a serious threat to public health worldwide. The present study indicated a high incidence of MBL producing *E. coli* (18.98%) and *K. pneumoniae* (21.08%) in different clinical samples. A previous study from an another tertiary care hospital in Nepal reported comparatively lower incidence of MBL producing gram negative bacteria (1.3%) in lower respiratory tract specimens [12]. However, a recent study from Nepal addressed the issue of increasing incidence of MBL producing *K. pneumoniae* (18.2%) in tracheal aspirate samples [13]. Several recent studies from other parts of Asia also demonstrated increasing incidence of MBL production in *Enterobacteriaceae isolates* [14–16]. In general, production of MBL in *Enterobacteriaceae* isolates currently follows an increasing prevalence pattern and the prevalence rate may vary greatly in different geographical areas and from institute to institute. In our hospital setting, extended spectrum beta-lactamases are prevalent in *E. coli* and *K. pneumoniae* isolates and there is a gradual rise in the use of carbapenems, which could be a major cause of MBL-mediated resistant.

The majority of MBL producing isolates of *E. coli* (53.56%) and *K. pneumoniae* (58.97%) were from patients admitted to ICU. The ICU has been described as a factory for creating, disseminating, and amplifying antimicrobial resistance [17]. Among the four different sources of samples (urine, sputum, pus and blood), we observed highest incidence of MBL producing isolates in blood samples and lowest incidence in urine samples for the both pathogens. This observation also indicated the greater use of carbapenems in bloodstream infections in our setting.

Detection methods for carbapenemase production include MHT, double disc test, blood agar combined disc assay, PCR amplification, and DNA sequencing [18]. CLSI recommends the MHT as a phenotypic confirmatory test for detection of carbapenemase production in *Enterobacteriaceae* isolates with elevated MICs for carbapenems or reduced inhibition zones in disc diffusion susceptibility testing, due to its acceptable sensitivity and specificity for carbapenemase detection. However, the sensitivity and the specificity of this test for detecting low-level MBL production are not known [9]. In this study, out of 80 *E. coli* and *K. pneumoniae* isolates initially screened for carbapenemase production, 73 (91.25%) isolates exhibited fairly positive results in MHT, whereas 7 (8.75%) isolates exhibited weakly positive results in MHT. Different studies also reported the occurrence of weakly positive results for the MHT in the detection of MBL producing *Enterobacteriaceae* [19, 20].

On the other hand, the combined disc test performed to detect MBL production was found to be highly sensitive (100%) as this test showed positive results with all the isolates of *E. coli* and *K. pneumoniae*, which were initially screened for carbapenemase production by disc diffusion test. The difference in the zone diameter was fairly more than 4 mm for each of isolate tested by combined disc test. Franklin et al*.* [11] also reported 100% sensitivity of combined disc test in the detection of MBL in gram negative bacilli. These results suggest that combined disc test can be used as a convenient method for detection of MBL producing *E. coli* and *K. pneumoniae* isolates in clinical laboratory on a daily basis. Conversely, the combined disc test would not detect class A carbapenemases such as GES, KPC and SME, while the MHT would do so. Besides, molecular detection of carbapenemase genes is an interesting alternative but remains costly and requires substantial expertise.

MBL producing bacterial isolates can confer resistance to carbapenems and all beta-lactam agents except aztreonam although coexistence of other resistance mechanisms such as AmpC type beta-lactamases or ESBLs render them resistant to aztreonam [20]. Likewise, all the isolates of *E. coli* and *K. pneumoniae* with MBL production in the present study were found to be resistant to all three carbapenems (imipenem, meropenem and ertapenem). These isolates also exhibited a high level of resistance to the penicillins, the third and fourth generation cephalosporins, cephamycin, and aztreonam, as well as to the beta-lactam/beta-lactamase inhibitor combination tested in the study. These findings are similar with other reports [21, 22], whereas in few reports MBL producing *Enterobacteriaceae* isolates were found to be susceptible to various carbapenems as well as to piperacillin/tazobactam by disc diffusion testing [23].

In this study, we observed that MBL production in *E. coli* and *K. pneumoniae* isolates was not always associated with elevated MIC values for the different carbapenems tested and these MIC values varied greatly among the MBL producing isolates. This variation in the MIC values for the different carbapenems may be influenced by several factors, such as the type and the expression of the carbapenemase enzyme, the bacterial species and the presence of other resistance mechanisms (e.g., Extended spectrum and AmpC beta-lactamases, reduced permeability and/or efflux pumps) [24]. Among the MBL producing isolates, the lowest MIC value for imipenem, meropenem and ertapenem were 2 μg/mL, 3 μg/mL and 8 μg/mL respectively for *E. coli* and 2 μg/mL, 2 μg/mL and 6 μg/mL respectively for *K. pneumoniae* isolates*.* The highest MIC value of >32 μg/mL was observed for all the three carbapenems in 12.82% MBL producing isolates of *K. pneumoniae.* Conversely, 7.3% of MBL producing isolates of *E. coli* showed the highest MIC value of >32 μg/mL only for ertapenem. Carbapenem MICs for carbapenemase producers may vary within a broad range of values, and even lay within the susceptibility range, as defined by either the current CLSI breakpoints. Indeed, such low levels of resistance to carbapenems have often been observed in *Enterobacteriaceae* producing carbapenemases of different types [25]. Therefore, any *Enterobacteriaceae* isolate with reduced susceptibility to carbapenems either by disc diffusion or MIC testing should be tested for carbapenemase production.

It has been observed that carbapenemase producers are usually associated with many other non beta-lactam resistance determinants, which give rise to “MDR and pandrug-resistant” isolates [26]. We also observed 100% MBL producers as “MDR” as well as 75.60% of *E. coli* and 71.79% of *K. pneumoniae* isolates producing MBL were “pandrug-resistant”. These results highlighted a potential threat to hospitalized patients by limiting the therapeutic options. Among the MBL producing isolates, comparatively lower resistant rate was for levofloxacin than ciprofloxacin, amikacin and gentamicin. Different in vitro studies reveal that tigecycline and colistin are the only agents with consistent activity against MDR or pan-resistant MBL-producing isolates [27]. In the present study, all the MBL producing isolates of *E. coli* and *K. pneumoniae* were also sensitive to tigecycline and colistin by disc diffusion test and their MIC values were within the susceptible range*.* Among these isolates**,** the MIC values for colistin ranged from 0.125 - 0.5 μg/mL and for tigecycline ranged from 0.125 - 2.0 μg/mL. However, the emergence of colistin and tigecycline resistance in *Enterobacteriaceae* is particularly menacing the future treatment options for bacterial infections [28, 29].

## Conclusions

Our findings showed MBL production in a considerable number of *E. coli* and *K. pneumoniae* isolates with MDR and pandrug-resistant phenotypes. In the majority of hospitalized patients, MBL production in *E. coli* and *K. pneumoniae* is associated with increased mortality, morbidity and cost. Therefore, early detection of MBL producing *E. coli* and *K. pneumoniae* isolates has become indispensable for clinical microbiology laboratories. In absence of molecular detection techniques, the combined disc test provides a sensible choice for phenotypic detection of MBL production and can be implemented in clinical laboratory on a daily basis. In addition, routine surveillance of MBL producing bacteria is crucial for establishing appropriate empirical antimicrobial therapy and restraining their spread in hospital environment.

## Abbreviations

MBL: Metallo-beta-lactamase
*E. coli*: *Escherichia coli*
*K. pneumoniae*: *Klebsiella pneumoniae*
EDTA: Ethylenediaminetetraacetic acid
MHA: Mueller-Hinton agar
CLSI: Clinical Laboratory Standards Institute
MIC: Minimum inhibitory concentration
MHT: Modified Hodge test
ICU: Intensive care units
MDR: Multidrug-resistant.
